# A Novel Drug‐Loaded Porous Parylene Electrode for Targeted Anti‐Inflammatory Therapy and Hearing Preservation in Cochlear Implantation

**DOI:** 10.1002/adhm.71593

**Published:** 2026-08-16

**Authors:** Chi‐Chieh Chang, Ying‐Chang Lu, Yu‐Ming Chang, Yen‐Hui Chan, Hong‐Yu Yan, Yi‐Shan Lee, Pei‐Hsuan Lin, Cheng‐Yu Hsieh, Chuan‐Jen Hsu, Tien‐Chen Liu, Hsien‐Yeh Chen, Chen‐Chi Wu

**Affiliations:** ^1^ Department of Otolaryngology‐Head and Neck Surgery National Taiwan University Hospital Taipei Taiwan; ^2^ Department of Chemical Engineering National Taiwan University Taipei Taiwan; ^3^ Department of Otolaryngology‐Head and Neck Surgery Taichung Tzu Chi Hospital Buddhist Tzu Chi Medical Foundation Taichung Taiwan; ^4^ Department of Otolaryngology‐Head and Neck Surgery National Taiwan University Hospital Hsin‐Chu Branch Hsinchu Taiwan

**Keywords:** cochlear implantation, dexamethasone, drug delivery, otoprotection, parylene

## Abstract

This study investigates the feasibility and therapeutic effectiveness of a novel drug‑loaded parylene electrode prototype (PEP) designed to reduce inflammation and hearing loss associated with cochlear implantation (CI). The porous PEP was fabricated using vapor‑phase sublimation and deposition, producing an interconnected structure with 20–30 µm pores and mechanical flexibility suitable for intracochlear placement. Dexamethasone was incorporated into the device (Dex/PEP), and in vitro characterization using ELISA and spectrophotometry demonstrated controlled first‑order release kinetics. Guinea pigs underwent microsurgical implantation of PEPs, with accurate positioning confirmed by micro‑CT imaging. Auditory outcomes were assessed through auditory brainstem response (ABR) measurements, and cochlear tissues were examined histologically to evaluate inflammatory cell infiltration and cytokine expression. The Dex/PEP device exhibited biosafety comparable to traditional silicone electrodes and provided sustained drug release. Animals receiving Dex/PEP showed significantly improved ABR thresholds at 1 and 4 weeks compared with blank PEP controls (*p* < 0.05), indicating reduced inflammation‑induced hearing loss. Histological analysis further revealed diminished inflammatory cell infiltration and markedly decreased TNF‑α expression at the round window membrane (*p* = 0.001). These findings support the drug‑loaded PEP as a promising targeted delivery platform for reducing cochlear inflammation and preserving hearing following CI.

## Graphical abstract

A porous parylene‐coated electrode loaded with dexamethasone was developed to reduce inflammation after cochlear implantation. In guinea pigs, the device provided sustained drug release, maintained biocompatibility, improved auditory brainstem response thresholds, and reduced inflammatory cell infiltration and TNF‐α expression. These findings suggest enhanced hearing preservation and reduced implantation‐related cochlear damage.

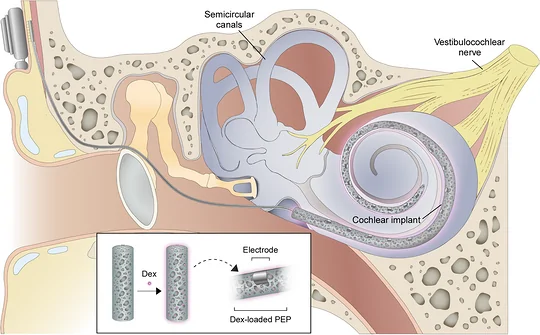

## 1 Introduction

Hearing loss is a common sensory impairment and the third most prevalent health problem in adults, after cardiovascular disease and arthritis [1]. The World Health Organization (WHO) estimates that 430 million people worldwide currently suffer from disabling hearing loss, a number projected to rise to 700 million by 2050 [2]. Beyond causing communication difficulties, hearing loss can negatively affect academic and work performance and increase the risk of social isolation, emotional distress, and dementia [3]. While hearing aids are the primary intervention, they offer limited benefit to individuals with profound hearing loss. For these individuals, cochlear implantation (CI) is an effective solution [4]. CI bypasses damaged inner ear structures and directly stimulates auditory neurons, enabling sound perception [5].

A cochlear implant consists of external and internal components that communicate via transcutaneous radiofrequency transmission. The external processor captures and encodes acoustic signals, which are then transmitted to the implanted receiver. There, the signals are converted into electrical impulses that activate spiral ganglion neurons (SGNs) via an electrode array inserted into the tonotopically organized cochlea [6]. Anatomically, the cochlea consists of three fluid‐filled compartments: the scala vestibuli, the scala media, and the scala tympani. These compartments are separated by Reissner's membrane and basilar membranes [7]. During standard CI surgery, an electrode array comprising platinum‐iridium lead wires and platinum contacts embedded in a silicone carrier is inserted into the scala tympani via the round window (RW).

Despite the generally positive outcomes of CI, challenges persist. For instance, electrode insertion can damage the delicate structures of the inner ear, causing inflammation and fibrotic tissue formation [8]. This injury can lead to increased electrode impedance and loss of hair cells and SGNs, resulting in reduced electrical stimulation efficiency, loss of residual hearing, and ultimately decreased speech perception [9, 10]. Various strategies aim to reduce this injury. One approach is the local delivery of steroids, which reduces inflammation, protects hair cells and neurons, and minimizes fibrosis [11, 12, 13, 14, 15, 16]. However, traditional non‐porous silicone electrode arrays hinder the effective, targeted delivery of drugs. Their low surface area limits the adsorption and encapsulation of drugs, thereby reducing the efficient loading and sustained release of steroids into the cochlea [17, 18].

Our previous research focused on developing novel parylene scaffolds through a vapor sublimation and deposition process using a dynamic ice template [19, 20]. This technique enables the creation of porous, three‐dimensional structures that can deliver drugs locally [21, 22]. Building on this foundation, this study investigates the application of these parylene scaffolds in developing a prototype drug‐eluting CI electrode. We fabricated a porous, rod‐like parylene electrode prototype (PEP) that matches the dimensions of standard CI electrodes while facilitating rapid drug release. The primary objective is to demonstrate the feasibility of incorporating corticosteroids directly into the electrode architecture during the fabrication process to achieve sustained release. This platform is intended for future integration with established conductive electrode arrays, enabling combined electrical stimulation and localized pharmacological therapy. Such an approach aims to enhance therapeutic outcomes by effectively delivering anti‐inflammatory agents to the inner ear, thereby preserving hearing following surgically induced trauma in in vivo models.

## 2 Methods

### 2.1 Development of the Pep

A porous, rod‐like PEP with an outer diameter of 0.6 mm and a length of 3 mm was fabricated through a solidification process followed by vapor sublimation and chemical vapor deposition (CVD) [23]. In brief, a precursor solution consisting of 1% polyurethane and 2% carboxymethyl cellulose (CMC; Showa Chemical, Japan) was prepared and cast into a polydimethylsiloxane (PDMS) mold. The solution was then solidified into rod‐shaped ice templates via a cryogenic process. For dexamethasone‐loaded PEPs (Dex/PEPs), dexamethasone (250 mg/mL; Sigma‐Aldrich, USA) was added to the formulation and processed under identical conditions.

The ice templates were then transferred to the deposition chamber of a custom‐built CVD system. During the sublimation and deposition stages, dichloro‐[2,2]‐paracyclophane (GALXYL, Italy) was sublimated at 50°C and pyrolyzed at 670°C to generate reactive quinodimethane monomers. These monomers then deposited and polymerized onto the sublimating ice template maintained at 0°C. Argon gas (25 sccm) was used as the carrier gas. The vapor composition and process progression were monitored in real time using a residual gas analyzer (RGA, HAL RC 511, Hiden Analytical, UK).

The three‐dimensional surface profiles and exterior morphology of the PEP were characterized using a confocal laser scanning microscope (VK‐9500; Keyence, Japan) and a Nova NanoSEM (FEI, USA), respectively. The pore size distribution of the PEP was quantified using mercury intrusion porosimetry (AutoPore IV 9520, Micromeritics, USA) across a pressure range of 0.1–60 000 psi.

### 2.2 In Vitro Characterization of Drug Release Kinetics

The release kinetics of the PEP were assessed using both Brilliant Blue FCF dye and dexamethasone (Cat. #D4902, Merck, Germany). To analyze dye release, stained PEPs were immersed in phosphate‐buffered saline (PBS) at 37°C. Absorbance was measured at 630 nm at the following time intervals: 0.5, 1, 3, 4, 8, and 16 h [24]. For the dexamethasone release assay, 3 mm Dex/PEP segments were incubated in 15 µL of artificial perilymph (1.3 mM CaCl_2_, 1.8 mM MgCl_2_, 5.4 mM KCl, 137 mM NaCl, 5 mM glucose, and 5 mM HEPES) at 37°C. The artificial perilymph volume (15 µL) was strategically selected to approximate the reported perilymphatic volume of the guinea pig cochlea [25]. This direct volume alignment ensures that the in vitro concentration kinetics closely emulate the localized concentration dynamics expected in vivo. Samples were collected at 1, 3, 5, 25, and 30 h, and the dexamethasone concentration was quantified using a Dexamethasone Forensic ELISA Kit (Cat. #101519, Neogen Corporation, USA) by measuring absorbance at 650 nm [26]. Standard curves were established for both markers to ensure accurate quantification.

### 2.3 Animal Models and Microsurgical Implantation

Guinea pigs (250–300 g) were acclimated for 4–5 days before the initiation of experiment. A trans‐tympanic approach was utilized for the insertion of the 3‐mm PEP, modeling the dimensions of established commercial matrices (e.g., MED‐EL [27] and NuSil [15]). This minimally invasive trajectory bypassed the need for a traditional bulla ototomy, thereby preserving natural middle ear pressure mechanics and eliminating bone dust generation. Under general anesthesia induced by isoflurane, a microsurgical procedure was performed to implant the PEP using an operating microscope (OPMI Pico; Carl Zeiss, Germany). The animals were secured in a lateral position with the head tilted toward the contralateral side. To facilitate insertion, a 3‐mm PEP was introduced through the tympanic membrane using a dissecting probe. The device was then carefully advanced into the posterosuperior quadrant of the middle ear and through the round window into the cochlea. Post‐operative monitoring confirmed that the transient tympanic membrane micro‐perforations created during the procedure healed spontaneously within 1–2 weeks without observable infectious complications.

To establish the surgical protocol and verify electrode placement via micro‐CT (SkyScan 1076, Belgium) [28], a subset of PEPs was pretreated with uranyl acetate for 1 week to enhance radiopacity. For subsequent biosafety and efficacy evaluations, all animals were implanted with non‐pretreated PEPs. All experimental procedures were conducted in strict accordance with the guidelines of the Institutional Animal Care and Use Committee of the National Taiwan University Hospital.

### 2.4 Auditory Function Assessments

Auditory brainstem response (ABR) thresholds were measured using an evoked potential detection system (Smart EP 3.90; Intelligent Hearing Systems, USA) to evaluate auditory function at baseline and at 1 and 4 weeks after surgery [29]. ABRs were evoked using broadband clicks and tone bursts (16 and 32 kHz) at varying intensities. The stimuli were delivered at a repetition rate of 20 Hz, and 400 sweeps were averaged for each measurement to optimize the signal‐to‐noise ratio. ABR waveforms were recorded via subcutaneous needle electrodes: the active electrodes were positioned at the vertex and the ipsilateral retroauricular region, while the ground electrode was placed on the animal's back.

### 2.5 Biosafety Assessment and Control Group Preparation

To evaluate the biosafety of the PEP, the induced foreign body response was compared against a silicone electrode scaffold of identical dimensions using the same surgical approach. The silicone electrode was fabricated via a solution‐casting technique. Polydimethylsiloxane (PDMS; SYLGARD 184, Dow Inc., USA) was degassed and infused into a glass capillary with a 600 µm internal diameter. Following thermal curing, the glass mold was mechanically fractured to isolate the silicone elastomer. For this comparative group, ABR thresholds were recorded at 1 and 4 weeks post‐implantation to assess functional biocompatibility.

### 2.6 Histopathological and Immunohistochemical Analysis

Cochleae were harvested 4 weeks post‐surgery and immediately fixed in 4% paraformaldehyde. The specimens were decalcified in 0.5 M EDTA (Cat. No. E177, VWR International LLC, USA) and subsequently embedded in paraffin. Mid‐modiolar sections (7 µm thick) were stained with hematoxylin and eosin (H&E) for histopathological examination by experienced veterinary pathologists. For immunohistochemical (IHC) analysis, sections were stained with a primary antibody against tumor necrosis factor‐α (TNF‐α, #ab1793, Abcam, UK) to quantify the inflammation response [30]. The primary antibodies were detected using a fluorochrome‐conjugated secondary antibody (Alexa Fluor 594, Invitrogen, USA), and the cell nuclei were counterstained with Hoechst 33342 (Cat. No. H3570, Invitrogen, USA). All histological and fluorescent images were captured using an ApoTome.2 imaging system (Carl Zeiss, Germany).

### 2.7 Statistical Analysis

All statistical analyses were conducted using GraphPad Prism 9 software (GraphPad Software Inc., USA). Depending on the number of independent variables, the data were analyzed using one‐way or two‐way analysis of variance (ANOVA), followed by a Tukey's post hoc multiple comparison test. Quantitative data are presented as mean ± standard deviation (SD). Statistical significance was pre‐defined at *p* < 0.05.

## 3 Results

### 3.1 Characterization of the PEP for Animal Studies

**FIGURE 1:**
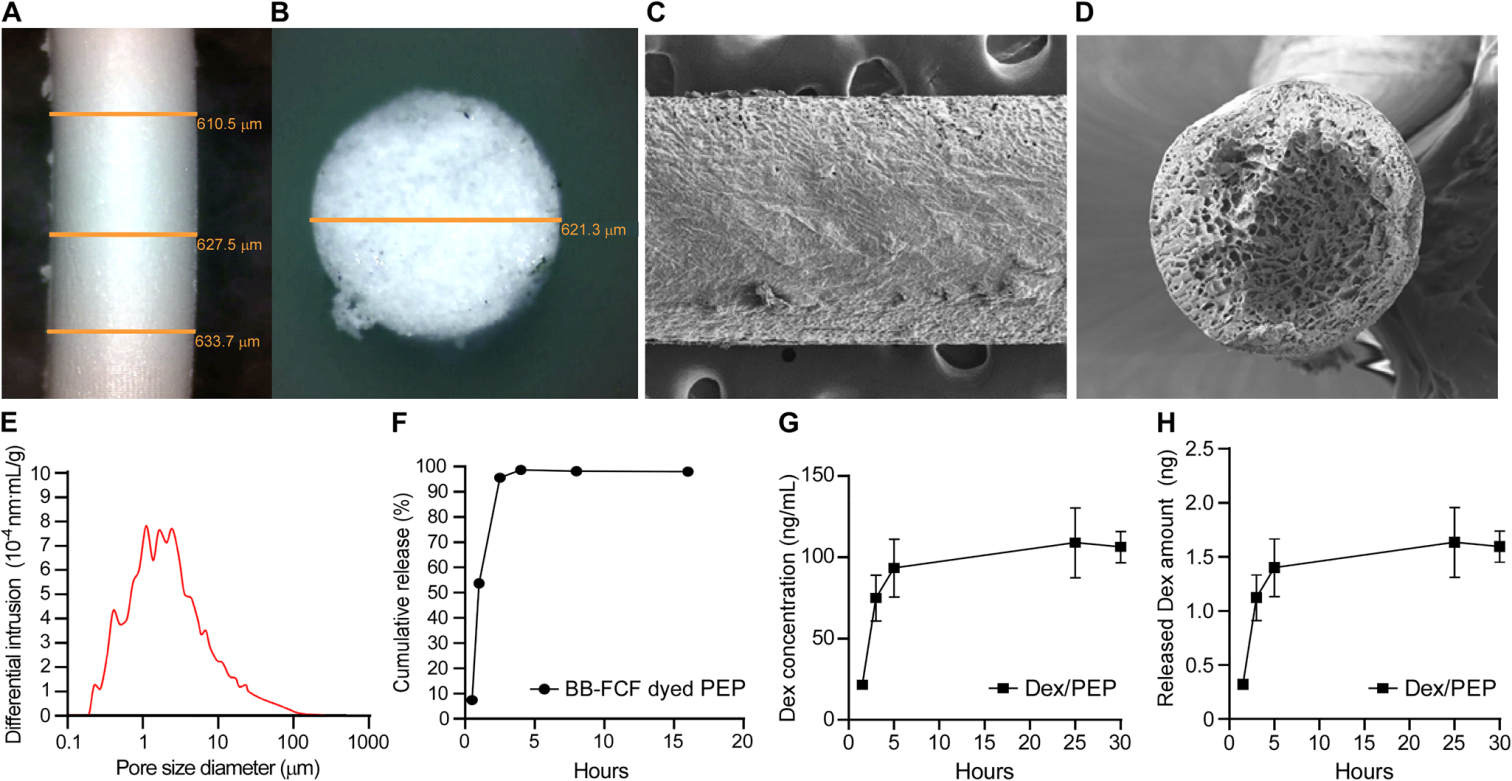
Development and physicochemical characterization of the parylene electrode prototype (PEP). This figure illustrates the structural architecture, surface morphology, and drug release kinetics of the novel porous PEP designed for cochlear implantation. (A and B) Macroscopic images confirm the maintenance of precise dimensions (approximately 0.6 mm outer diameter) during fabrication. (C and D) Scanning electron microscopy (SEM) reveals an interconnected porous network with pore sizes ranging from 20 µm to 30 µm. (E) Pore size distribution analyzed via mercury intrusion porosimetry over a pressure range of 0.1–60 000 psi. (F–H) In vitro release kinetics of Brilliant Blue FCF (BB‐FCF) and dexamethasone. (F) Cumulative release percentage (%) of BB‐FCF over time, demonstrating passive diffusion dynamics. (G) Dexamethasone concentration (ng/mL) measured at the indicated time points within a physiological volume (15 µL) of artificial perilymph. (H) Amount of dexamethasone released (ng) at each time point, calculated relative to the estimated 15 µL total perilymphatic fluid volume of the guinea pig cochlea.

Utilizing our established fabrication protocols [23], we developed a porous, rod‐shaped PEP designed to emulate the dimensions of a clinical CI electrode. Depositing parylene onto a sublimating ice template resulted in an interconnected porous architecture that can encapsulate non‐volatile therapeutic agents for localized release. Optical imaging confirmed consistent, circular, rod‐shaped morphology with an average diameter of 623.9 ± 12.0 µm (Figure 1A,B). Scanning electron microscopy (SEM) revealed a homogeneous distribution of irregularly shaped, interconnected pores ranging from 20 µm to 30 µm in size, confirming the presence of a continuous porous network (Figure 1C,D). Mercury intrusion porosimetry revealed an exceptionally high porosity of 96.84%, characterized by a predominant population of micrometer‐scale pores and an average pore size of 7.98 µm (Figure 1E). This highly porous architecture reduces resistance to mass transport by providing shortened diffusion pathways and high permeability.

### 3.2 In Vitro Drug Release Kinetics

The drug delivery capabilities of the PEP were evaluated using Brilliant Blue FCF dye and dexamethasone. Both molecules were uniformly distributed within the porous network and released primarily via passive diffusion. The PEP exhibited rapid dye release, achieving over 50% cumulative release within 1 h and completing release within 4 h (Figure 1F). Similarly, dexamethasone concentration increased rapidly during the initial 5 h of incubation in artificial perilymph and subsequently reached a stable plateau sustained between 5 and 30 h (Figure 1G,H). The measured dexamethasone concentration ranged from approximately 90–118 ng/mL throughout this plateau phase, which corresponds to a released dexamethasone amount of approximately 1.3–1.8 ng within 15 µL artificial perilymph volume.

### 3.3 Microsurgical PEP Implantation in Guinea Pigs

**FIGURE 2:**
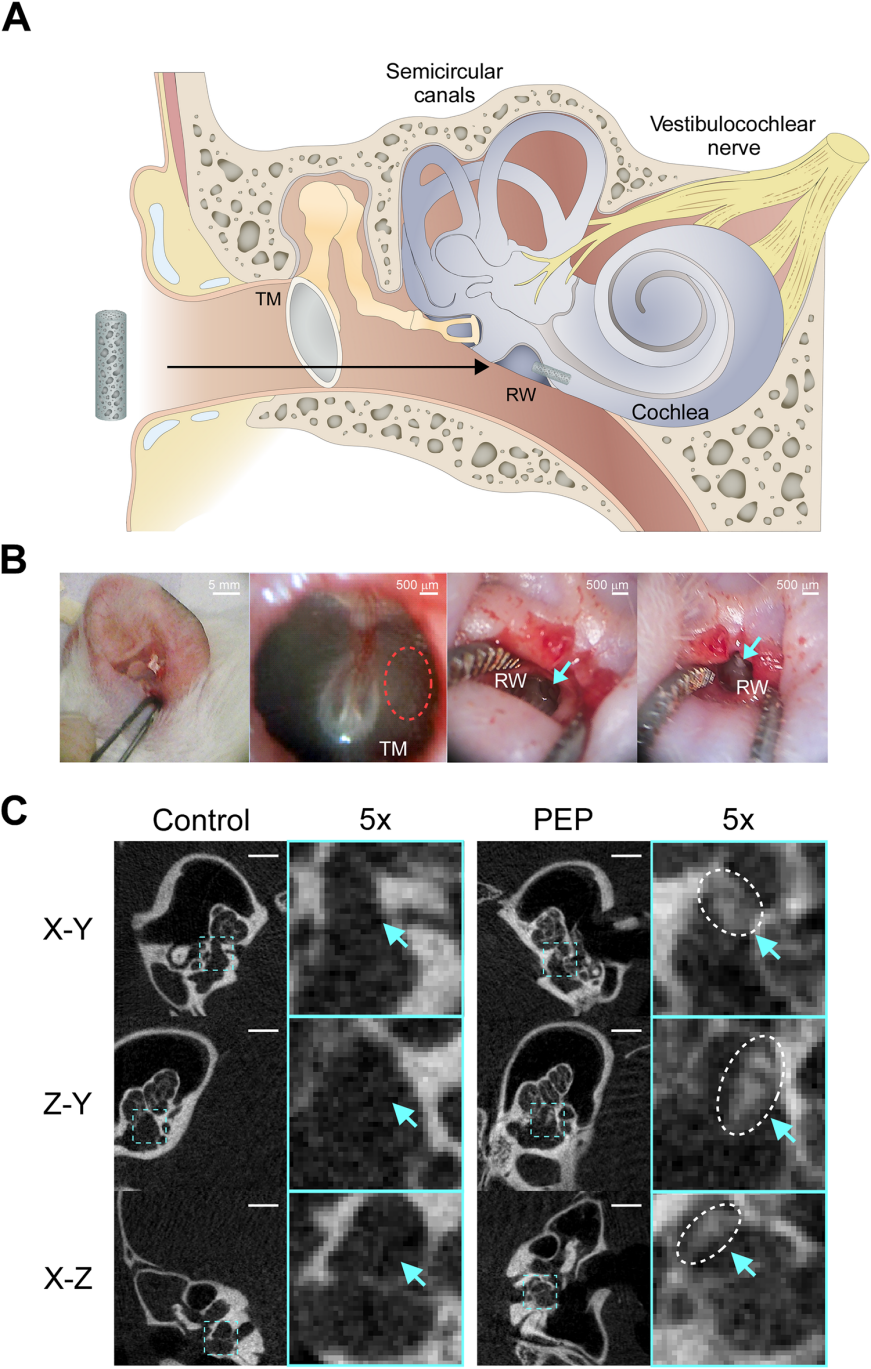
Microsurgical technique and verification of intracochlear PEP implantation. This figure details the surgical approach and confirmation of intracochlear placement in guinea pigs. (A) Schematic illustrating the surgical route: PEP insertion through the tympanic membrane (TM) and into the cochlea via the round window (RW). (B) Otoscopic visualization confirms the introduction of the PEP into the RW niche. Cyan arrows indicate the precise entry point at the RW. (C) Orthogonal planes (X–Y, Y–Z, and X–Z) of three‐dimensional micro‐CT reconstructions verify the precise intracochlear placement of uranyl acetate‐treated PEPs compared to control ears. Dashed white ovals and cyan arrows highlight the radiopaque PEP positioned within the scala tympani. Scale bars = 1 mm.

A specialized microsurgical technique was developed for intracochlear PEP implantation (Figure 2A). After puncturing the tympanic membrane with a dissecting probe, the 3‐mm PEP was inserted through the RW into the scala tympani. Successful positioning within the RW niche was verified via otoscopic visualization (Figure 2B). Furthermore, three‐dimensional micro‐CT reconstructions of uranyl acetate‐treated PEPs confirmed precise intracochlear placement compared to control ears (Figure 2C).

### 3.4 Biosafety Evaluation of the PEP

To evaluate biosafety, the foreign body response elicited by the PEP was compared to that of a silicone electrode scaffold of identical dimensions using the same surgical approach. Three control groups were included to distinguish surgical trauma from device‐related effects: a pre‐surgery group, a sham group (involving only the perforation of the tympanic membrane), and an insertion‐only group (involving acute insertion of the PEP followed by immediate withdrawal). ABR thresholds were measured at 1 and 4 weeks post‐implantation. Following the procedures, the sham group exhibited minimal auditory damage, with an ABR threshold of 35 dB SPL for click stimuli (*n* = 2, data not shown). The insertion‐only group demonstrated the baseline of acute mechanical surgical trauma, presenting thresholds of 43.3 ± 4.1 dB SPL for click, 21.7 ± 10.8 dB SPL for 16 kHz, and 40.8 ± 7.4 dB SPL for 32 kHz. Both the long‐term implanted PEP and silicone electrode groups exhibited significant, yet statistically comparable, elevations in high‐frequency hearing thresholds relative to this insertion baseline. These parallel shifts reflect the unavoidable mechanical constraints of the animal model combined with standard foreign body encapsulation, confirming that the parylene matrix shares an identical functional biosafety profile with conventional clinical‐grade silicone.

**FIGURE 3:**
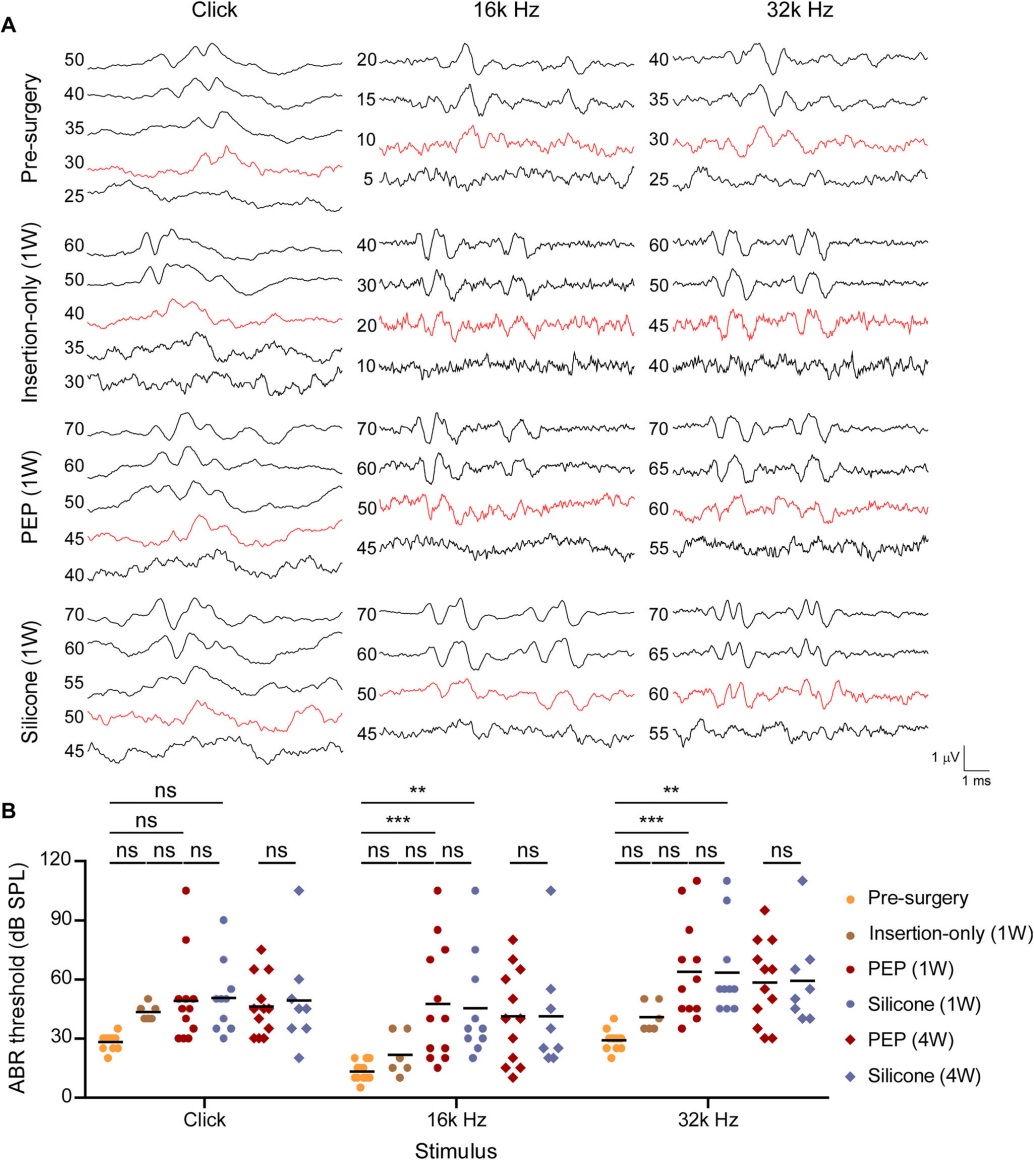
Hearing thresholds after insertion of PEP and silicone electrode into the guinea pig cochlea. Auditory brainstem response (ABR) thresholds were measured to evaluate hearing function using click and tone burst stimuli (16 and 32 kHz). (A) Representative ABR waveforms recorded pre‐surgery and at 1 week post‐insertion for the insertion‐only, the blank PEP and silicone electrode groups. (B) Quantitative comparison of individual hearing thresholds among three groups: pre‐surgery baseline (*n* = 12), insertion‐only (*n* = 6), blank PEP (*n* = 12), and silicone electrode implantation (*n* = 10 at 1 week; *n* = 8 at 4 weeks). The data demonstrate comparable auditory threshold shifts between the PEP and silicone groups, suggesting similar biosafety properties (two‐way ANOVA with Tukey's test; ** *p* < 0.01; *** *p* < 0.001).

Specifically, at 16 kHz, thresholds increased from a pre‐surgical baseline of 13.3 ± 4.9 dB SPL to 47.5 ± 29.7 dB SPL for the PEP group (*p* = 0.001) and 45.5 ± 26.7 dB SPL for the silicone group (*p* = 0.004). Similarly, at 32 kHz, thresholds increased from a pre‐surgical baseline of 29.2 ± 5.1 dB SPL to 63.8 ± 25.1 dB SPL for the PEP group (*p* = 0.001) and 63.5 ± 23.2 dB SPL for the silicone group (*p* = 0.002). These comparable results, analyzed via two‐way ANOVA with Tukey's test, suggest that the PEP possesses biosafety properties equivalent to those of conventional silicone materials used in clinical cochlear implants (Figure 3).

### 3.5 In Vivo Efficacy of Dexamethasone‐Loaded PEPs

**FIGURE 4:**
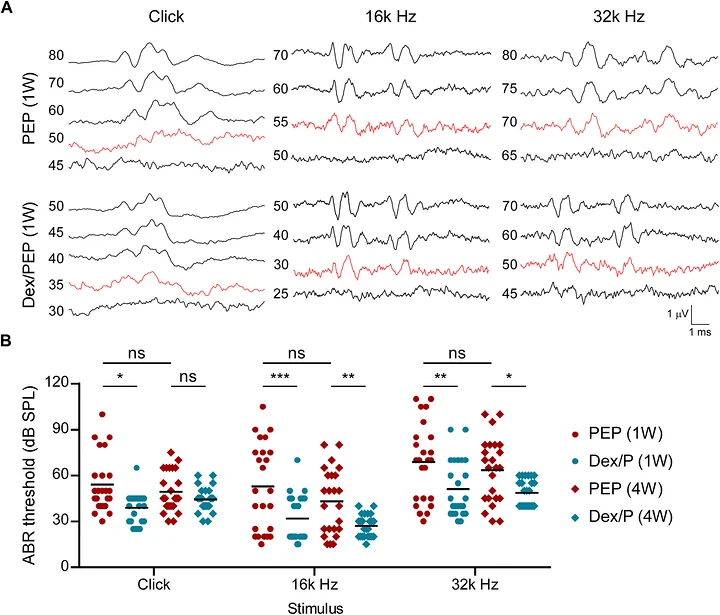
Otoprotective effects of dexamethasone‐loaded PEP (Dex/PEP) against surgical trauma. Auditory brainstem response (ABR) data demonstrating the protective effect of Dex/PEP on hearing function. (A) Representative ABR waveforms at 1 week post‐insertion showing improved hearing thresholds in the Dex/PEP group. (B) Quantitative comparison of hearing thresholds between the blank PEP and Dex/PEP groups (*n* = 24 per group). At 1 week, the Dex/PEP group showed significantly lower thresholds across all stimuli. At 4 weeks, the Dex/PEP group maintained a sustained protective effect at 16 and 32 kHz compared to the blank PEP group (Tukey's two‐way ANOVA; * *p* < 0.05; ** *p* < 0.01; *** *p* < 0.001).

To verify the therapeutic efficacy of drug‐loaded electrodes, PEPs preloaded with 0.001 mg/mL dexamethasone (Dex/PEP) were implanted and compared to blank PEPs. One week post‐surgery, the Dex/PEP group demonstrated significantly improved ABR thresholds across all tested frequencies compared to the PEP group. Specifically, click thresholds were 39.0 ± 9.8 dB SPL in the Dex/PEP group versus 54.2 ± 18.6 dB SPL in the PEP group (*p* = 0.016). At 16 kHz, the thresholds were 31.9 ± 15.2 dB SPL vs. 52.9 ± 28.5 dB SPL (*p* < 0.001), and at 32 kHz, they were 51.3 ± 18.5 dB SPL vs. 68.8 ± 25.2 dB SPL (*p* = 0.004). These findings indicate a robust early otoprotective effect facilitated by the dexamethasone payload (Figure 4A,B).

By 4 weeks post‐insertion, the blank PEP group exhibited partial threshold recovery, suggesting a degree of spontaneous restoration following the initial surgical trauma. In contrast, the Dex/PEP group continued to exhibit significantly lower hearing thresholds than the blank PEP group, maintaining a sustained protective advantage. At 16 kHz, Dex/PEP group thresholds remained lower at 27.1 ± 7.2 dB SPL compared to 43.1 ± 21.3 dB SPL in the PEP group (*p* = 0.009). Similarly, at 32 kHz, the Dex/PEP group recorded 48.8 ± 8.8 dB SPL versus 63.5 ± 21.1 dB SPL in the PEP group (*p* = 0.021; two‐way ANOVA with Tukey's test) (Figure 4B).

### 3.6 Histopathological and Immunohistochemical Analysis of the Cochlea

**FIGURE 5:**
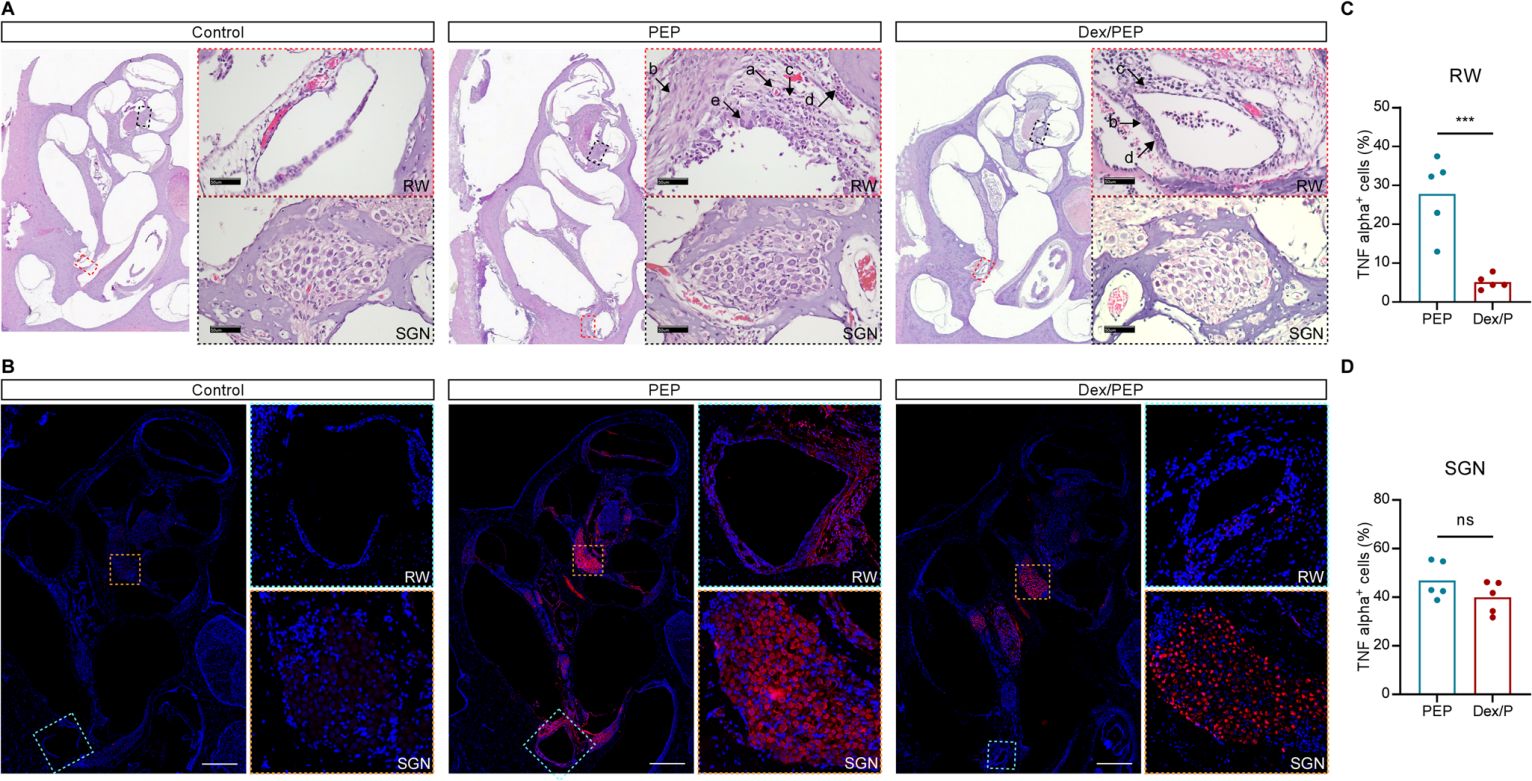
Histopathological and immunohistochemical assessment of inflammatory response. Anti‐inflammatory effects of Dex/PEP evaluated 4 weeks post‐surgery. (A) Hematoxylin and eosin (H&E) staining shows minimal neovascularization (arrows a), fibrosis (arrows b), and infiltration of mononuclear cells (arrows c), neutrophils (arrows d), and multinucleated giant cells (arrows e) in the blank PEP group, which were reduced in the Dex/PEP group. Scale bars = 50 µm. (B) Fluorescent immunohistochemistry (IHC) for TNF‐α (red) and Hoechst (blue) shows signals around spiral ganglion neurons (SGNs) and the round window membrane (RW). Scale bars = 200 µm. (C and D) Quantitative analysis confirms a significant reduction in TNF‐α expression at the RW in the Dex/PEP group (*** *p* = 0.001), while SGN expression showed only a slight, non‐significant reduction.

To evaluate the otoprotective effects of Dex/PEP against surgery‐induced trauma, cochlear tissues were examined histologically for lesions and inflammation 4 weeks post‐implantation. Histological analysis via H&E staining showed that blank PEP implantation resulted in increased infiltration of mononuclear cells, heterophils, and multinucleated giant cells, accompanied by fibrosis around the implant site (Figure 5A). These inflammatory and fibrotic manifestations were notably attenuated in the Dex/PEP group.

Fluorescent IHC analysis was employed to detect the expression of the broad inflammatory marker TNF‐α within the inner ear (Figure 5B). TNF‐α signals were predominantly localized around spiral ganglion neurons (SGNs) and the round window membrane (RWM). Quantitative analysis confirmed that TNF‐α expression at the RW was significantly lower in the Dex/PEP group than in the PEP group (5.1 ± 1.8 vs. 27.8 ± 9.9, *p* = 0.001), supporting a localized anti‐inflammatory effect. In contrast, TNF‐α expression surrounding the SGNs showed only a slight, statistically insignificant reduction in the Dex/PEP group (39.9 ± 6.7 vs. 46.8 ± 7.7, *p* = 0.166) (Figure 5C,D). These histopathological findings collectively demonstrate the effectiveness of anti‐inflammatory drug delivery and the protective benefits of the Dex/PEP application.

## 4 Discussion

This study introduces a novel drug‐loaded PEP designed to overcome the limitations of current anti‐inflammatory strategies in CI. The PEP demonstrated precise structural construction, mechanical durability, and efficient drug delivery capabilities through both in vitro and in vivo validations. By utilizing an established microsurgical technique, we achieved accurate PEP placement in a guinea pig model, verified by micro‐CT. Crucially, the PEP exhibited comparable to traditional silicone electrodes, which are the current clinical standard. The significant improvement in ABR thresholds observed in the Dex/PEP group underscores the potential of this technology to mitigate inflammation‐induced hearing loss. Furthermore, histopathological evidence confirmed the ability of the PEP to effectively deliver therapeutic agents and suppress the inflammatory response triggered by surgical trauma, with no indications of generalized middle ear infection or acute labyrinthitis in the adjacent sections.

The development of effective and safe CI electrode arrays necessitates a careful balance of material properties and design to ensure atraumatic insertion [31]. An ideal electrode array must provide high flexibility to navigate the cochlear duct and long‐term mechanical stability to prevent complications such as cable breakage or loss of the hermetic seal [32]. While silicone remains the primary backing material in contemporary CI electrodes, innovative polymers like parylene offer a unique opportunity to optimize electrode performance by enhancing both functionality and biocompatibility [33, 34, 35]. Local corticosteroid delivery is an increasingly vital strategy in this context, as these agents have been shown to reduce postoperative inflammation, minimize structural tissue damage, and protect delicate intracochlear elements [36, 37, 38, 39].

Various strategies have been developed to enhance corticosteroid delivery to the inner ear: (1) direct incorporation of drugs into the electrode carrier, (2) integration of pump‐connected delivery channels, and (3) application of polymer‐based surface coatings [40]. Regarding the first strategy, the non‐porous nature of standard silicone significantly limits its capacity for targeted drug delivery, as the low surface area restricts both drug adsorption and sustained release. In contrast, the second strategy utilizes cannulated electrode systems that allow for precise control of drug dosage and infusion duration. Recent studies indicate that intracochlear infusion via micro‐catheters, combined with a dexamethasone‐eluting electrode, effectively preserves residual hearing and reduces oxidative stress, thereby mitigating CI‐induced inflammation [41]. Prospective randomized controlled trials are currently evaluating the safety and efficacy of commercial dexamethasone‐eluting silicone arrays in human subjects (e.g., NCT06598059, NCT06424262). Preliminary findings and systematic reviews suggest that these drug‐eluting arrays effectively reduce post‐implantation electrical impedance and mitigate the inflammatory foreign body response [42, 43, 44].

While cannulated systems provide precise dosage control, the third strategy, surface coating, offers a substantially simpler configuration that eliminates the need for external components while enabling programmable release kinetics. Our Dex/PEP system demonstrates a distinct advantage over cannulated eluting systems specifically regarding dexamethasone delivery. Cannula‐based systems require the drug to be saline‐soluble, necessitating the use of dexamethasone phosphate. In contrast, our porous parylene framework allows for the delivery of dexamethasone in its base form, which diffuses more rapidly across tissue boundaries to achieve more efficient perilymphatic distribution [45]. In this study, the measured dexamethasone concentration within the physiological perilymph volume remained stable between 90 ng/mL and 118 ng/mL from 3 h to 30 h. This concentration profile successfully meets or exceeds the established target therapeutic window (50–100 ng/mL) documented to suppress acute inner ear inflammation [15]. These findings support the potent localized therapeutic potential of our porous parylene platform during the critical acute post‐implantation window.

Among current coating materials, parylene offers distinct clinical and engineering advantages over other biodegradable polymers such as polytrimethylene carbonate (PTMC) and polycaprolactone (PCL) reported in the literature [26, 46, 47, 48]. First, the superior flexibility and elasticity of parylene facilitate atraumatic insertion, ensuring optimal electrode placement by seamlessly conforming to the spiral anatomy of the cochlea [49]. Second, whereas biodegradable polymers undergo degradation that may compromise structural integrity and change local chemistry, parylene provides exceptional long‐term stability and chemical inertness within the in vivo environment (Table 1). This stability is vital for permanent implants like CIs, as it allows for the formation of highly conformal, protective coatings that serve as a robust, integrated platform for drug delivery.

**TABLE 1:** Comparison of cochlear implant (CI) electrode materials.

Feature	Silicone	PTMC	PCL	Parylene
Components	Poly(dimethylsiloxane) semicrystalline polymer	Polyester amorphous polymer	Polyester semicrystalline polymer	Poly(2‐chloro‐*p*‐xylylene) semicrystalline polymer
Structural Integrity	Highly stable but non‐porous, limiting drug loading	Integrity compromised during hydrolytic degradation	Loses mechanical robustness during degradation	Exceptional long‐term stability and chemical inertness in vivo
Porosity and architecture	Non‐porous; low surface area for drug adsorption	Typically non‐porous coating	Variable coating density	Interconnected porous network with 96.84% porosity
Drug delivery mechanism	Limited to slow, surface‐level diffusion	Release tied to polymer degradation rates	Hydrolytic degradation‐controlled release	Passive diffusion via first‐order kinetics; high permeability
Mechanical performance	High elasticity but bulkier carrier	High flexibility but transient integrity	Moderate flexibility; degradation affects fit	Excellent flexibility/elasticity for atraumatic insertion
Conformability	Standard bulk fit	Soft tissue conformity	High surface conformity	Highly conformal and protective coating for complex geometries
Primary advantage	Established clinical standard	Simple surface coating	Neutral degradation products	Multifunctional platform for acute and chronic protection

*Note*: Data were compiled exclusively from peer‐reviewed sources describing explicit material properties [55, 56].

Abbreviations: PCL, polycaprolactone; PTMC, polytrimethylene carbonate.

A critical future implication of this research is that the porous parylene scaffold serves as a compelling foundation for a dual‐phase, time‐controlled drug delivery system [50, 51], which is widely considered the “gold standard” for next‐generation CI electrodes. While our current PEP achieves the rapid‐release profile necessary to mitigate acute postoperative inflammation, long‐term CI success also depends on the sustained delivery of neurotrophic factors, such as brain‐derived neurotrophic factor (BDNF) or glial cell‐derived neurotrophic factor (GDNF), to prevent progressive degeneration of SGNs [52, 53, 54]. Because our fabrication process enables precise control over material porosity and the creation of multi‐layered structures, future iterations can leverage the versatility of parylene to encapsulate distinct therapeutic agents within separate reservoirs. For instance, a porous core could be utilized for fast‐releasing steroids, while a surrounding diffusion‐limiting layer provides long‐term neuroprotection. This adaptability overcomes the fundamental material constraints of traditional non‐porous silicone, positioning the drug‐loaded parylene electrode as a truly multifunctional platform for both acute tissue preservation and chronic neuroprotection.

The strengths of this study lie in the comprehensive characterization of the PEP, which demonstrates both excellent biocompatibility and high drug‐loading efficiency. The significant improvement in ABR thresholds observed in guinea pigs treated with dexamethasone‐loaded PEPs provides compelling evidence that this technology can effectively mitigate surgical trauma and improve functional outcomes. Importantly, the PEP is designed as a modular drug‐eluting scaffold that forms the structural basis of a complete cochlear implant electrode, with the capacity to incorporate conductive elements for electrical stimulation. Unlike conventional drug delivery coatings applied onto pre‐existing electrodes, the PEP represents a designable electrode architecture in which structural, pharmacological, and electrical functions can be integrated within a single platform. This design concept enables independent optimization of these components, providing a flexible strategy for enhancing current CI technologies through combined electrical stimulation and localized drug delivery. However, several limitations must be acknowledged. First, the surgical methodology imposes inherent translational constraints. The trans‐tympanic trajectory utilized herein diverges from the clinical gold standard of a postauricular bulla ototomy, which is necessary to attain optimal exposure of the round window niche for deep‐array insertions (e.g., advancing to the second cochlear turn). Furthermore, given that the requisite tympanic membrane perforation introduces a theoretical vector for middle ear infection, further studies must address this translational limitation by employing the standard postauricular paradigm, which will eliminate the infectious risk while facilitating a more comprehensive evaluation of deep intracochlear biomechanics under true human surgical vectors. Second, while the guinea pig model is a standard for auditory research, the findings may not be fully translatable to the complex anatomy and clinical requirements of human subjects. Additionally, the 4‐week follow‐up period, while sufficient for assessing acute inflammatory responses, does not provide data on the long‐term efficacy or potential chronic complications associated with the device. Consequently, future research utilizing larger animal models and, ultimately, human clinical trials is essential to fully validate the safety and long‐term performance of this promising multifunctional electrode platform.

## Author Contributions

**Chi‐Chieh Chang**: data curation, formal analysis, investigation, methodology, validation, visualization, writing – original draft, writing – review and editing. **Ying‐Chang Lu**: data curation, formal analysis, investigation, methodology, validation, visualization. **Yen‐Hui Chan**: investigation, methodology. **Yi‐Shan Lee**: investigation, methodology. **Pei‐Hsuan Lin**: validation, supervision. **Cheng‐Yu Hsieh**: validation, supervision. **Chuan‐Jen Hsu**: validation, supervision. **Tien‐Chen Liu**: validation, supervision. **Hsien‐Yeh Chen**: conceptualization, funding acquisition, investigation, supervision, validation, writing – review and editing. **Chen‐Chi Wu**: conceptualization, funding acquisition, investigation, methodology, project administration, resources, supervision, validation, visualization, writing – review and editing.

## Funding

The authors gratefully acknowledge funding support from the National Science and Technology Council of Taiwan (NSTC 110‐2314‐B‐002‐189‐MY3 and NSTC 112‐2221‐E‐002‐025‐MY3) and National Taiwan University Hospital (114‐SS0053).

## Conflicts of Interest

The authors declare no conflicts of interest.

## Data Availability

The data that support the findings of this study are available from the corresponding author upon reasonable request.
